# Recent Progress in the Topologies of the Surface Acoustic Wave Sensors and the Corresponding Electronic Processing Circuits

**DOI:** 10.3390/s22134917

**Published:** 2022-06-29

**Authors:** Mariya Aleksandrova, Dimiter Badarov

**Affiliations:** 1 Department of Microelectronics, Technical University of Sofia, 1000 Sofia, Bulgaria; 2 Department of Electronics, Technical University of Sofia, 1000 Sofia, Bulgaria; dbadarov@tu-sofia.bg

**Keywords:** surface acoustic waves (SAWs), SAW sensors, interdigital (IDT) electrodes, sensor processing circuits

## Abstract

In this paper, we present an overview of the latest achievements in surface acoustic wave (SAW) sensors for gas or liquid fluid, with a focus on the electrodes’ topology and signal processing, as related to the application of the sensing device. Although the progress in this field is mainly due to advances in the materials science and the sensing coatings, the interdigital (IDT) electrodes’ organization is also an important tool for setting the acoustic-wave-distribution mode, and, thus, for improvement of the SAW performance. The signal-conditioning system is of practical interest, as the implementation of the SAW, as a compact and mobile system is dependent on this electronic circuit. The precision of the detection of the SAW platform is related not only to the IDT electrodes’ geometry but also to their location around the sensing layer. The most commonly used architectures are shown in the present paper. Finally, we identify the needs for the future improvement of these prospective sensors.

## 1. Introduction

The surface acoustic wave (SAW) is a mechanical wave with a specific frequency range propagating through the surface of a piezoelectric substrate or thin film. It is generated from an electrical signal applied to the input pairs of comb-shaped electrodes, called an interdigital transducer (IDT), relying on the direct piezoelectric effect. After traveling like a mechanical wave through the piezoelectric material, it is, in turn, converted back into an electrical signal with the help of the output pairs of similar IDT electrodes, which are usually symmetric to the input pair. The input- and output-transducing electrodes are characterized by their periodicity and sizes related to the frequency of the SAW [1]. The whole device topology is organized in such a way that the vibrational energy is concentrated on the top (surface) of the piezoelectric substrate/coating, and it is not distributed in the vertical direction in depth, in contrast to the bulk acoustic wave (BAW) devices, using the volume of the materials for spreading the wave [2]. According to the available experimental studies, the performance of SAW devices, as different types of sensors, is better in the frequency range from hundreds of kilohertz to a few gigahertz [3]. Following the design methodology, which is well-elaborated for the SAW filters in telecommunication, it is known that propagation of the SAW at higher frequencies, with minimal attenuation and dielectric losses, requires smaller features (width and pitch) of the IDT [4], which makes the sensors based on the SAW effect compact.

**Figure 1:**
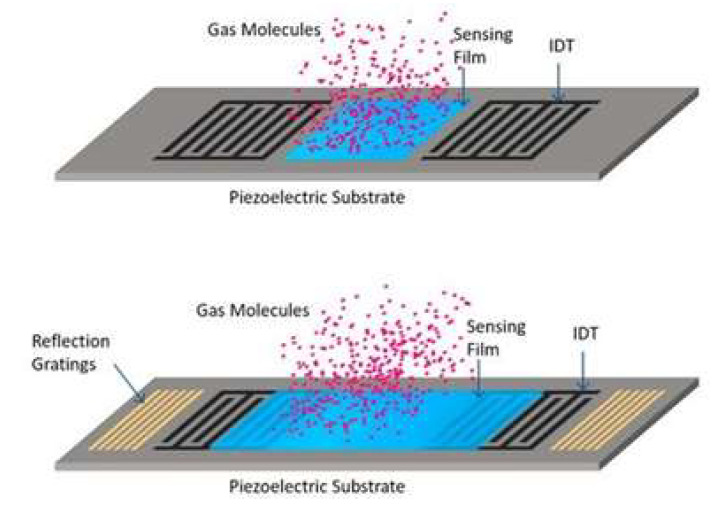
Common view of a SAW element with a piezoelectric substrate and sensing coating, located in between the IDTs (**upper**) and over the IDTs (**lower**) [9].

Fabrication of the SAW transducers is based on the thin metal films’ growth, by standard vacuum-deposition methods and direct- or reverse (lift-off)-lithography patterning, to shape the IDTs. In most of the cases, low-cost wet-chemical etching can be used as a final step of the photolithographic process [5]. If additional piezoelectric coating is needed in between or over the IDT electrodes, which is also formed by conventional processes such as vacuum RF sputtering, chemical vapor deposition (CVD), or spray coating (if the coating is organic) [6]. In this way, the large-scale production of the SAW elements is made compatible with the CMOS technology, except if the design is not based on the piezoelectric-crystal substrate. Then, a combination of microelectromechanical (MEMS) and CMOS technology is required to successfully integrate the SAW element and the integrated electronic circuits for signal-data processing [7]. In any case, the overall cost for fabrication of the SAW-based sensor devices is relatively low, as compared to other sensors relying on more precise and expensive microfabrication processes [8]. A common view of the SAW element with a piezoelectric substrate or coating is shown in Figure 1.

Small changes in the density of the matter on the path of the wave cause great changes in the propagation-surface conditions, making the magnitude, phase, or frequency of the output signal different as compared to the input signal’s parameters. This property of the SAW elements is widely used for a variety of sensor applications. Based on the type and degree of change of the SAW parameters, the degree of variation of the stimulus can be estimated, causing a change in the surface conditions. One of the possible direct uses of SAW sensors is the detection of pressure change, as it relies on the piezoelectric effect, so there is no need for additional sensing coating [10,11,12,13]. It is possible that the SAW serves as an accelerometer or gyroscope [14,15]. One of the most common approaches to increasing the pressure sensitivity of the SAW element is to fabricate it on a backside-etched silicon wafer, via the deposition of a thick piezoelectric film, by transferring a single piezoelectric crystal piece and bonding it to the silicon membrane [16]. The process is fully compatible with MEMS-fabrication technology. To increase the linearity of the pressure sensor, some researchers have replaced the flexible membrane with a flexible console device (beam or cantilever), such as a substrate for the SAW element [17].

The selection of a piezoelectric material with temperature dependence on the piezoelectric response makes possible the application of the SAW element as a temperature sensor as well. The temperature stability or instability depends on the cut of the piezoelectric crystal or the crystallographic orientation of the piezoelectric films. For example, it is well-known that AT-cut quartz is characterized by a temperature coefficient of resonance frequency change (TCF) close to zero in a broad temperature range (from 10 °C to 50 °C). Therefore, a SAW element based on such a substrate is not appropriate to work as a temperature sensor. However, a material such as LiNbO_3_ with a 128° Y–X cut demonstrated strong temperature dependence of the piezoelectric coefficients. In the range between 50 °C and 200 °C, the TCF showed a linear relationship with temperature, which was used for temperature detection with a satisfactory accuracy of better than ±1% [18,19]. Similar behavior was exhibited by LiTaO_3_, ZnO, and AlN materials [20,21], but they differed in their sensitivity and upper temperature-detection limit, which was determined by the Curie temperature of the substances, e.g., from 120 °C for ZnO to approximately 500 °C for AlN. In all cases, a negative TCF was observed. For better linearity of the TCF, the SAW-based temperature sensor is realized with two SAW resonators situated on the same substrate but having different orientation angles to each other, used after the calculation procedure only, so the ratio of the resonators’ wavelength should be set for accurate temperature measurement [22]. When the piezoelectric film is in question instead of the substrate, to guarantee the independence of the temperature measurement for the thermal properties of the substrate where the film grows, a substrate with strong thermal insulating properties is used, such as a sapphire, for example. These results suggest that a temperature compensation in the SAW element is needed for all other sensing applications to eliminate the temperature effects.

**Figure 2:**
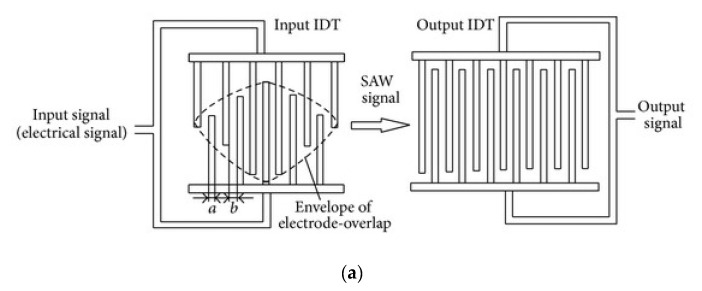

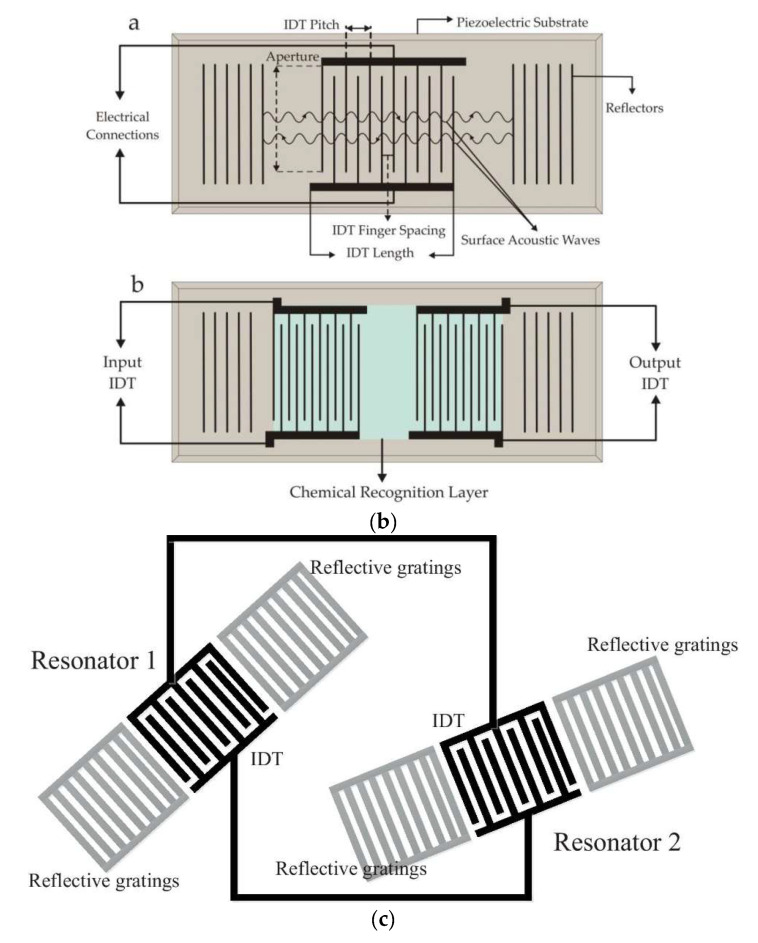
Common view of the SAW element for application as a: (**a**) force sensor [27]; (**b**) gas- or biosensor [28]; (**c**) temperature sensor. Reprinted with permission from Ref. [29]. 2020, Ling Li et al.

When additional sensing coating is added on the top of the IDT electrode, or between them, it is for the possible adherent attachment (adsorption, or absorption) of analytes with different origins, such as toxic gases, biological substances, humidity, etc. Then, the sensing mechanism is due to the mass loading, and it can be related to the change of the analyte’s concentration [23]. Changes in mass and viscosity at the biosensitive layer can be detected by recording changes in the acoustic-wave properties such as velocity, attenuation, resonant frequency shift, or time delay. Such a structure has been widely investigated for sensing and fluidic applications in advanced lab-on-chip complex devices [24,25]. Acoustic-wave sensors are able to monitor not only mass or density changes, but also changes in the Young modulus, viscosity, and dielectric and conductivity properties, wirelessly and in real-time [26]. These sensors can be used to detect small biomolecules by selectively binding to a thin-film adsorber for the detection of pathogens and viruses in a complex media of biological fluid, in which other molecular species are present together with the analyte. Depending on the fluid type (gas or liquid), the sensor is classified as an “electronic nose” (e-nose) or an “electronic tongue” (e-tongue). Figure 2 shows a common view of the SAW element, for application as a pressure sensor (Figure 2a), gas- or biosensor (Figure 2b), and temperature sensor (Figure 2c).

Nanotechnologies and the nanomaterials are strongly implemented in the SAW-based chemical sensors. Recently, gold nanorods and silver nanocubes have been synthesized for the potential volatile organic compounds (VOCs)-sensing coatings grown on SAW transducers [30]. The sensor is characterized by a high resolution in the ppb levels and the poor humidity dependence of the sensing performance. A 2D g-C_3_N_4_@TiO_2_ hybrid nanocomposite has been developed for NO_2_ SAW-based sensors [31]. Despite the longer response and recovery time, the sensor exhibited much higher sensitivity (in some cases more than 10 times) compared to earlier SAW NO_2_ sensors with thin metal-oxide sensing films.

The temperature sensitivity can be problematic, when the SAW device is not used as a temperature sensor. It can result in signal-profile deterioration, due to the temperature instability of the central frequency of the SAW structure. The velocity of the propagation of the acoustic waves is affected by the charges induced by the thermal expansion. The coupling-factor parameter is defined to reflect the change in SAW velocity, and it is related to the free surface-wave velocity and the velocity on the metallized surface [32]. The value of the coupling factor determines the bandwidth and the energy loss in the zone between the transducers. At the materials science level, this parameter is associated with the defects and irregularities of the piezoelectric substrate or film, causing wave scattering and energy dissipation. It is also related to the crystallographic orientation of the piezoelectric crystal and can be controlled by the cut angle and situation of the IDTs, to form a wave-traveling axis coinciding with the preferable direction of the propagation for a certain cut. The temperature coefficient of the delay is introduced to give information about the SAW-sensor behavior (frequency shift) due to a temperature change, and it is also dependent on the spatial orientation of the crystal planes and the crystal’s cut. According to the concrete application and the expected operational conditions, e.g., temperature variations in a specific range, for an operational frequency, and with acceptable losses, a suitable substrate should be selected, combining a high coupling factor and low temperature coefficients of expansion or delay.

Devkota et al. have prepared a review devoted to discussing SAW technique advances in sensing chemicals that can be in a liquid or a gaseous phase, considering the wave-distribution mode [9]. Possible reasons for signal attenuation, including changes in the propagation rate of the acoustic waves and other factors causing loss of information, have been taken into account as being related to the substrate type and its elastic properties. The thickness, flatness, smoothness, and mechanical strength of the substrate, for the variety of sensing layers and IDT materials, have been pointed out as possible tools for tuning the basic sensor characteristics and neutralizing the detection instability, due to factors such as humidity, temperature, and strain. Mujahid and Dickert have reviewed the influence of the different angle cuts of the crystals, when selecting piezoelectric substrates for specific SAW applications, such as gas or liquid biosensors [28]. Among the typical angles that have been highlighted are 36° Y-X for LiTaO_3_, 64° Y-X and 128° Y-X for LiNbO_3_ and quartz, etc. Different approaches, such as host–guest compositions, organic compounds, metal oxide nanofilms and biological receptors, have been proposed to affect the sensing layer’s hydrophility, mode of acoustic-wave propagation, limits of detection, and other sensing characteristics of interest. In another review by Mujahid et al., a comparison between the quartz-crystal microbalance and the SAW has been made in terms of sensor performance, again with a focus on the selection of the piezoelectric substrate with a specific cut and its importance for the temperature dependence of the line frequency [33]. A similar review has been authored by Panneerselvam et al., who also developed a thesis about the simulation and modeling of the SAW with an application as an e-nose, using the delay line and resonator structure of the sensor [34]. 

As a general conclusion, the focus in most of the reported research and review papers has been put on the nanomaterials being incorporated as sensing coatings and the fabrication processes. Two topics have been poorly discussed: (1) how the topology of the IDTs of the SAW-based chemical sensors affect the sensing characteristics, according to the specific applications; and (2) how the signal from the SAW is further processed by a suitable electronic circuit. Therefore, the present paper intends to fill this gap by providing more information about the design aspect of the SAW topology, together with the sensing electronics for fluid-sensing applications.

## 2. Effect of the IDTs Topologies on the Sensing Characteristics of SAW-Based Chemical Sensors, According to the Specific Applications

**Figure 3:**
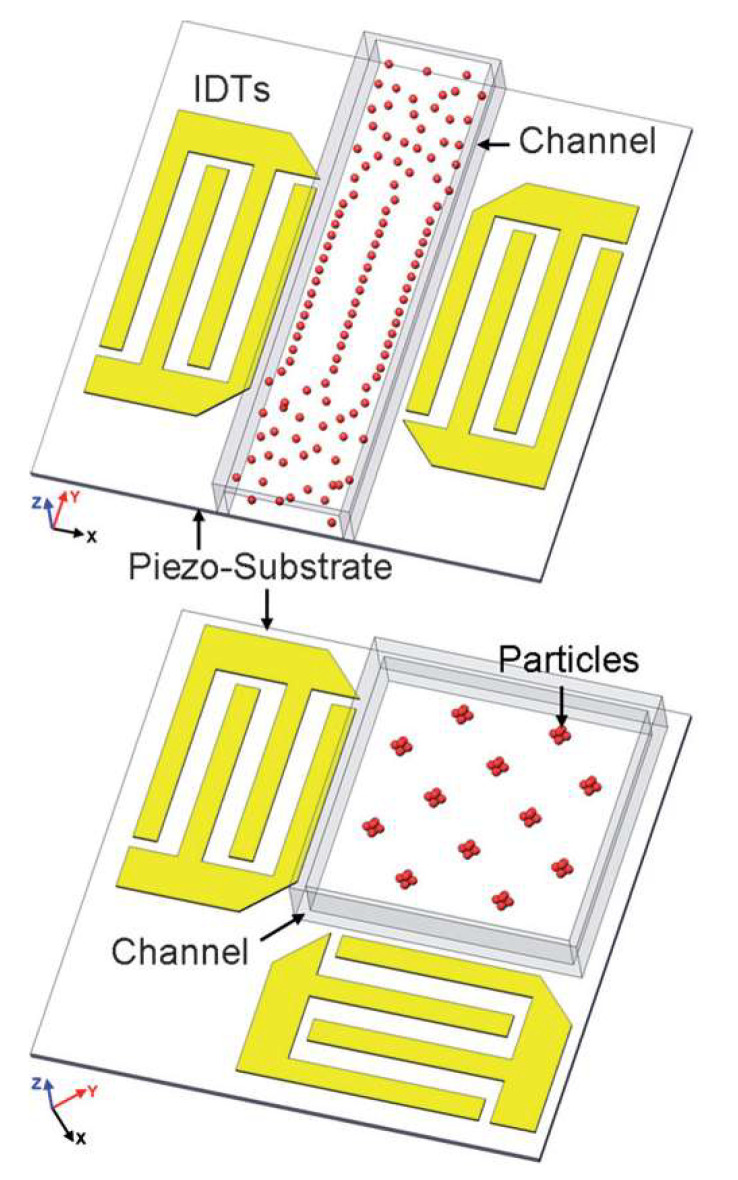
Topology of the IDTs gaining sorting of the atoms or molecules of interest. Reprinted with permission from Ref. [35]. 2009, Jinjie Shi et al.

The design of a SAW device, in particular for IDTs and back reflectors, is possible if the fingers height, width, period, and length of the comb electrode are known. The pattern is directly related to the resonance frequency of the SAW detector, so it is responsible for the sensitivity (resolution) of the measurements with SAW, although the selectivity and response time are determined by the features of the analyte-recognition films. The first design in Figure 3 helps to control the acoustic-wave propagation, allowing analytes to flow as a vapor flux or liquid fluid. The second design in Figure 3, with the square-shaped active zone, has been proposed for standing-wave generation, which can be used in addition as a sorting approach to capture, preferentially, only specific analytes and, thus, enhance the selectivity of the process.

In the first case, with the parallel IDT electrodes, a liquid media (solution) containing microparticles propagated in the space formed between the electrodes similar to a flow in a channel. The flow under pressure was first stabilized, and then the signal to the electrodes was switched on. Thus, two similar acoustic waves are generated opposite to each other, until they reach the boundary with the flow. There, because of an interference phenomenon, a standing SAW is formed, resulting in the modulation of the liquid-flow pressure in such a way that when the maximum pressure amplitude takes place, the particles are concentrated in the middle of the channel and their concentration can be measured as related to the parameters of the standing SAW. When the minimum pressure amplitude takes place, longitudinal waves driving the flow were dominant in the channel, and the microparticles are moved with the flow. In this way, the flow rate of the analyte can also be calculated. If the flow rate is fast, and the precision of the measurement risks being compromised, then the flow must be slowed or blocked for a period of time, corresponding to the response time of the measurement devices. Then, the standing SAW should be able to “capture” the microparticles for a certain time (determined by the topology of the IDTs, considering the flow rate). Such a function was performed by the orthogonal IDTs. It is worth mentioning that the angle of the pattern can be different than 90°, and this is an additional parameter of the SAW-device geometry for control of the sensor precision, by controlling the duration of the standing wave.

**Figure 4:**
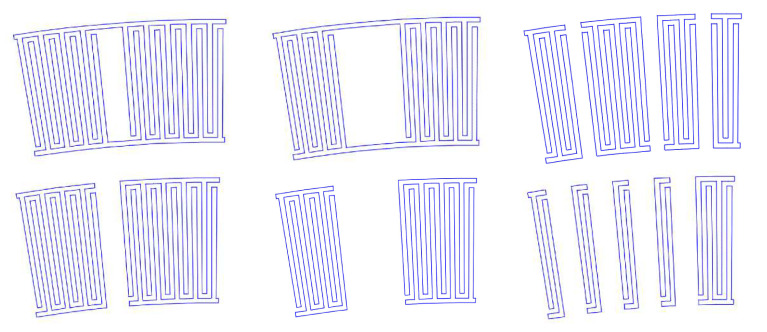
Selected view of segments from the ring-shaped SAW with and without common bus [36].

The circular topology, although not typical for the SAW, could also be optimized in terms of frequency characteristics (Figure 4).

To increase the selectivity of the SAW sensor, the period, length, and width of the electrodes should be changed, usually by making the structure asymmetric or irregular in the periodicity of its pins. The bandwidth of the device is inversely proportional to the number of electrode pairs, the reflections of the signal strongly increase. It was found that such effect can be reduced by using split electrode buses. Although the increased number of splits reduced the waste energy from reflections, rejecting the secondary modes in the amplitude-frequency characteristics and suppressing more efficiently the noise, the sensitivity tends to become lower. By selective removal of pins, this effect can be exhibited to a different extent, leading to a trade-off between the noise susceptibility and sensitivity and the design complexity. It has been found that the common bus assists in keeping the distribution of the acoustic wave outside the electrode-converting zone. The removal of a specific number of pins within the length of the IDTs, changing the periodicity of the electrodes, results in a narrowing of the bandwidth and an enhancement of the main signal’s peak for the target frequency, suppressing the harmonic distortions. Such a type of topology is known in the fluidic sensor for mixing fluids and producing a directional fluid stream with an enhanced diffusion rate, due to the focused SAW generation.

**Figure 5:**
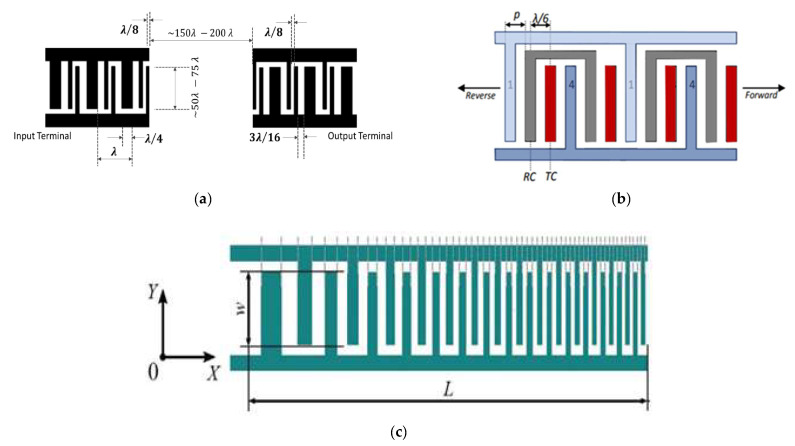
Modification in the period of the IDT: (**a**) split configuration; (**b**) floating-electrode configuration. Adapted with permission from Ref. [38]. 2017, Y.Q. Fu et al. [38]; (**c**) delay-line configuration. Adapted with permission from Ref. [39]. 2018, Dame Fall et al.

Irregular IDT configurations, which can be split or floating, have been applied to reduce reflection of the SAW toward the periphery of the substrate, out of the zone of the sensing-layer location [37]. The splitting period is related to the wavelength (respectively to the frequency) and can be calculated by sub-dividing the wavelength (Figure 5a). It is intended to suppress specific orders of harmonics, by keeping the ratio between the frequency and the width of the electrode fingers. The floating pattern means a specific number of electrodes in the sequence of fingers are to be disconnected from the common bus. In this way, higher working frequencies can be achieved without the need to decrease the width of the electrode fingers (Figure 5b). The delay line from Figure 5c is characterized by the gradually changing dimensions of the IDTs along the length of the line. The structure allows frequency modulation and a variety of available bandwidths, which is a very useful property for controlling the microfluidic flows in biosensors.

The negative effect caused by the strip-electrode structures, such as a decrease in the total wave-energy level, is controlled by a reasonable number of splits that are additionally combined with the proper selection of a substrate, having corresponding coupling factor and anisotropic properties, which keeps the balance between the low degree of energy reflection and the high basic-mode amplitude. The design is usually developed in such a way that the physical distance between the transducers is taken into account together with the delay time and phase. An optimum was found in the number of pairs, grating fingers, substrate thickness and aperture. The best case reported in terms of a frequency characteristic of the signal consists of 25 pairs of fingers with an aperture of 25λ (λ is central wavelength), when the number of grating fingers on each reflector is at least 10 [40]. The greater the number is, the higher the frequency of the signal. They can be split many times, and to suppress the reflections the variable aperture of the main fingers and the grating fingers are applied along the length of the structure. A typical example is a structure with three splits, to a set of 45, 25, and 35 pairs with an aperture of 25λ, 25λ, and 40λ, respectively, and grating fingers of 5, 10, and 15.

**Figure 6:**
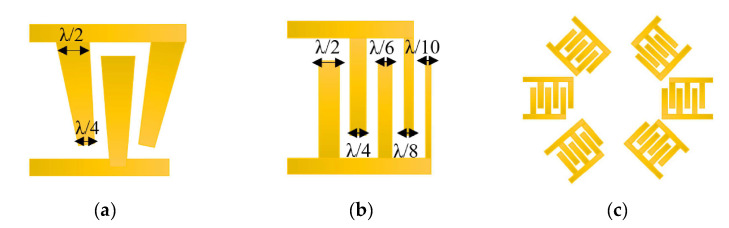
Non-conventional IDT electrodes designed for particle sorting: (**a**) slanted device; (**b**) device with variable in fingers width; (**c**) multiple-round forming device [41].

It was proven that the introduction of inclination angle, instead of the parallel fingers of the IDT electrodes, results in a highly selective separation of particles to a distance greater than λ/4, despite the size of the particles (Figure 6a When compared to the conventional SAW, the device from Figure 6b works at higher frequencies. The principle of increasing the distance between the fingers, with the number of fingers themselves, leads to expanding the frequency band, thus increasing the flexibility in operation with a variety of substances. Figure 6c depicts a focused SAW, which allows for the activation of a very small functional area (the middle cross-point of the waves’ path, when each SAW device is paired with the corresponding neighboring structure across. The effect is multiplying the amplitude of the wave, making the device sensitive and improving its work with gases or fluids at a lower power supply.

## 3. Processing of the SAW Signal by a Suitable Electronic Circuit

Modern sensor devices are often built like multisensory arrays intended for complex gas-mixture analyses. In addition, for better accuracy of detection, each of the main SAW lines should preferably work in differential mode, comparing its output signal with a reference channel that is not exposed to the analyte. In this way, temperature effects’ compensation, or impedance matching, can also be realized.

The signal-conditioning circuit has to measure precisely the change in the resonant frequency of the sensor. There are several different methods for signal conditioning, which are selected based on the self-resonant frequency of the sensor, the measurement time, and the measurement precision. There are two main methods: time-based and frequency-based [42]. The time-based method uses an oscillator with the sensor as a resonator and a precision frequency counter to measure the resonant frequency. This method is useful for sensors with a self-resonant frequency of several megahertz. It also requires a relatively longer measurement time for high precision. For sensors with a self-resonant frequency of several hundred megahertz, we can still use the time-based method, but the measurement of the resonant frequency will be indirect. The signal from the oscillator will be converted down with the use of a local oscillator and mixer, and the resultant frequency will be measured [43]. The frequency-based method is preferred for high self-resonance frequency sensors, when a short measurement time is required. In the frequency-based method, the sensor is connected as a load to a Voltage Controlled Oscillator (VCO), and a Phase-Locked Loop (PLL) system is built to change the VCO frequency based on the phase relation between the input and output signals of the SAW sensor.

**Figure 7:**
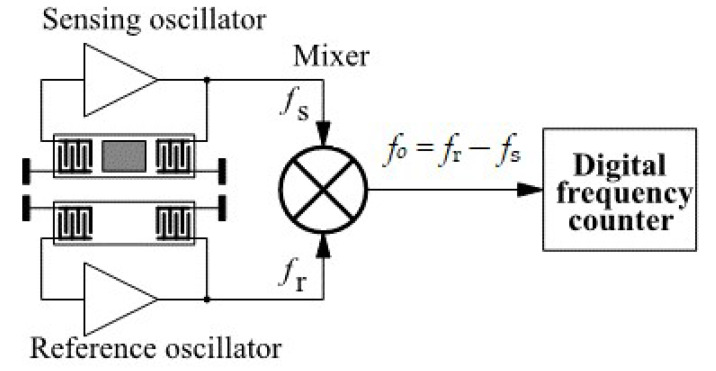
Two-channel SAW sensor with a frequency mixer [44].

A method for compensation of the frequency change due to temperature and other factors is needed, when the SAW sensor is used with a special gas-sensitive layer for the measurement of gas concentrations. Such a configuration is presented in Figure 7.

The circuit consists of two identical SAW sensors. The upper sensor has the gas-sensitive layer and is called “sensing”, while the lower sensor is without such a layer and is called “reference”. Every sensor is connected to a separate oscillator circuit. The output signals of the two oscillators are applied to an RF mixer, which generates the frequency difference between the two signals. This signal, after low-pass filtering, is applied to the frequency counter, which measures the frequency difference between the two channels. With this technique, the temperature and other changes in the resonant frequency are symmetrical in the reference and the sensing channels and do not produce a frequency change at the output of the mixer. At the same time, the frequency change due to the concentration of the measured gas is present only in the sense channel and is registered by the frequency counter at the output of the mixer [44].

**Figure 8:**
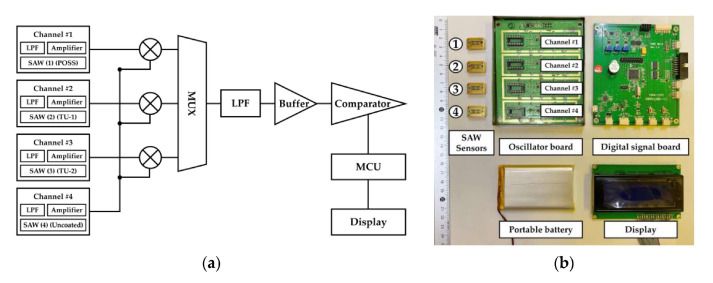
Portable multichannel sensor system with SAWs: (**a**) common schematic view; (**b**) image of the realized printed circuit board [45].

A similar principle has been used in complex sensor systems where multiple analytes should be detected, therefore, multisensor arrays should be designed. The difference between the single and multiple sensing systems is in the presence of a multiplexor for switching between the separate channels (Figure 8). The system works in differential mode, comparing the measurement of a reference device with some of the other active channels. The system is equipped with a low-pass filter (LPF) and an amplifier, which at the same time plays the role of a buffer for impedance matching between the conversion and the computing parts, ensuring power conditioning and minimizing energy loss. The system was organized into two printed circuit boards, called the oscillator board (containing the SAWs and the battery power supply) and the digital-signal processing board (containing the rest of the electronic circuits).

**Figure 9:**
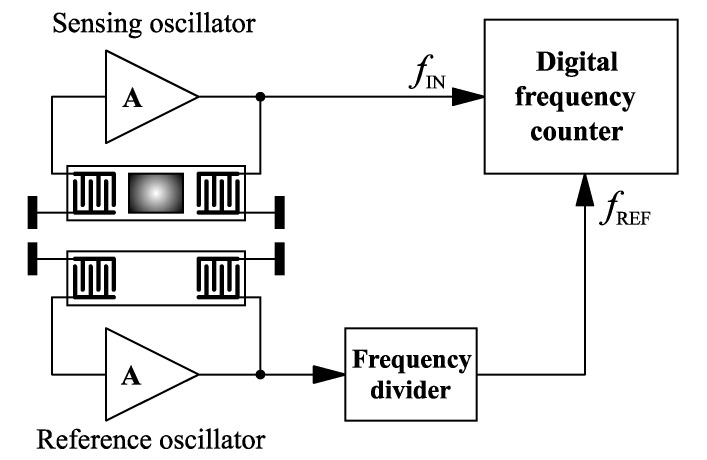
Two-channel SAW sensor with a frequency divider [46].

In the cases of sensors with lower resonant frequencies, generally less than 1 MHz, the concept of a mixer for frequency conversion is too complicated and does not lead to high accuracy. In such cases, the most useful approach is to include the sensor in a classical oscillator circuit, for example a Colpitts oscillator, and the output frequency is to be directly measured with a high-accuracy frequency counter (Figure 9). In this configuration, either one port or two port sensors can be used. If a compensation channel for temperature and other variations is needed, a second reference oscillator with a sensor without a sensitive layer can be added. The signal from the reference oscillator, after a frequency division, can be used as a reference signal for the frequency counter, canceling out the frequency changes caused by the different sources, except the measured parameter.

**Figure 10:**
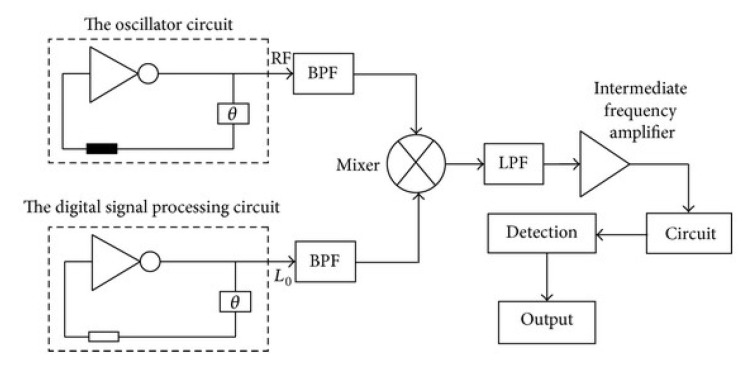
Dual-channel structure of a SAW-based sensor device [27].

Although the basic electronic circuits for SAW signal processing have similar conceptual design and main building blocks, sometimes there are additional elements according to the specific application. For example, Figure 10 shows that there are band-pass filters (BPF) after the reference and measured oscillating devices, before the mixer. In addition, after the mixer, there is a low-pass filter (LPF), an amplifier, and a shaping circuit. This approach enhances the frequency stability, therefore, the resolution of the measurement and the overall performance of the sensor are very high, because these additional components directly affect the stability.

**Figure 11:**
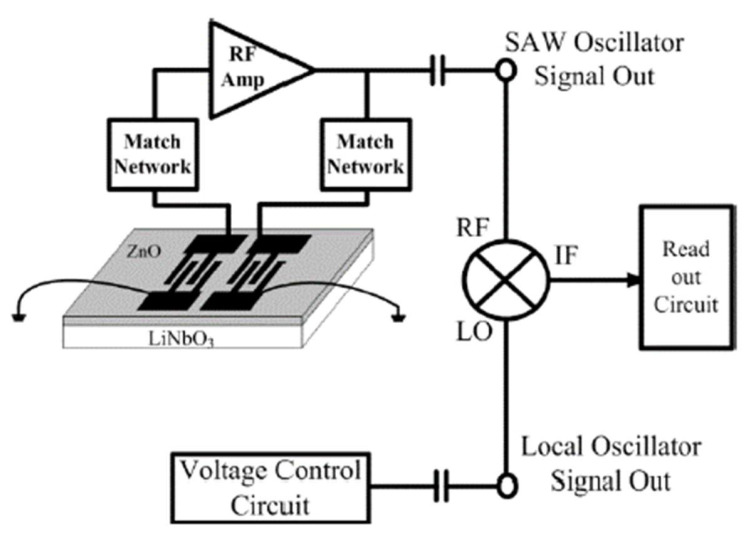
Circuit for processing the signal from the SAW-based sensors [47].

As is shown in Figure 11, sometimes there is an amplifier before the mixer as well as matching circuits rather than filters. The frequency-to-voltage converter consists of a phase-locked loop (PLL) and a differential amplifier. The output voltage is proportional to the input frequency necessary for the converter. The output voltage is then converted to a digital signal in the reading circuit.

**Figure 12:**
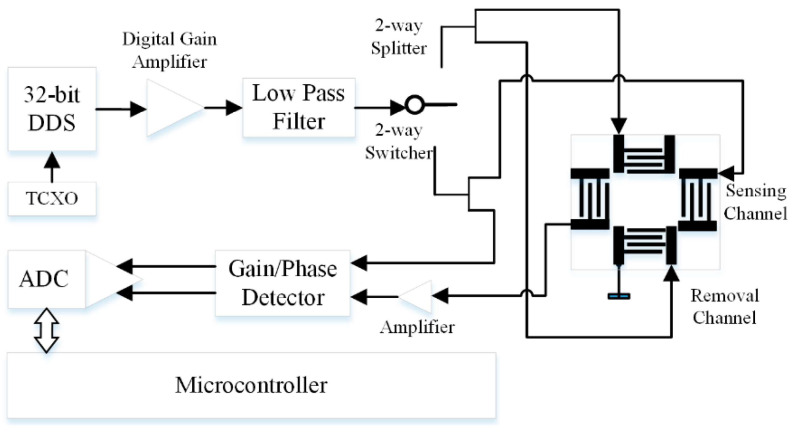
Electronic circuit of complex SAW configuration with direct digital synthesizer (DDS) [48].

Figure 12 shows a suitable solution for the realization of the signal-conditioning system, by using a compact direct digital synthesizer (DDS) as a portable version of the conventional large-size supplementary equipment for SAW testing. The bridge-similar IDT structure of the SAW sensor requires gain control, a phase-shift or amplitude-decay measuring unit, and data processing in terms of analog-to-digital conversion. The gain/phase detector works in comparator mode for the testing and reference channels.

## 4. Conclusions

We have proposed an overview of the most widely applied IDT topologies for SAW sensing devices, which includes additional function such as particle sorting. We have discussed the electrodes’ geometry and the situation on the substrate as factors influencing the frequency characteristics and the suppression of harmonic distortions. Specifics of the signal-processing electronics for a simple single-SAW device and for a complex multiple-SAW array system have been given. Concrete practical applications have been commented on, in terms of the electrodes pattern and electronic blocks for signal conditioning.

Although the SAW approach for gas detection has been pointed out as sensitive, stable, and fast, it seems that the problem with the recovery time has not been solved yet, which may affect the reproducibility. This limitation has not been discussed broadly in the research, and there is no obvious connection between the electrodes’ topology and the recovery time. The incomplete and relatively slow recovery has been ascribed to the sensing mechanism and the principle of interaction between the analyte molecules and the gas-sensing coatings. In order to improve this sensor characteristic, a microheater can be integrated on the backside of the piezoelectric substrate (or layer). Its operation will boost the desorption of the analyte molecules and, therefore, the mass unloading effect on the sensing layer. The topology of the microheater is crucial for the proper distribution of the heat, in a way to prevent response degradation with the temperature. If not designed and located appropriately, it may change the temperature coefficient of the frequency of the piezoelectric substrates or the temperature coefficient of the expansion of the IDT electrodes and sensing coatings. Thus, this issue will be the focus of the researchers for future optimization of the SAW devices also in terms of metal films and multilayer metallization, suitable for mounting processes and packaging of the sensor. On this basis, it will be developed a technology process for vacuum deposition of coatings, needed to bond the samples, which is intended to be realized in the laboratories on micro/nanoassembling and micropackaging, belonging to the biomechatronics section of CoE on Mechatronics and clean technologies.

## Data Availability

Not applicable.
